# Adverse events in Chinese human immunodeficiency virus (HIV) patients receiving first line antiretroviral therapy

**DOI:** 10.1186/s12879-020-4878-2

**Published:** 2020-02-19

**Authors:** Lili Dai, Bin Su, An Liu, Hongwei Zhang, Hao Wu, Tong Zhang, Ying Shao, Jianwei Li, Jiangzhu Ye, Shaoli Bai, Xiaoling Guo, Lijun Sun

**Affiliations:** 1 Center for Infectious Diseases, Beijing Youan Hospital, Capital Medical University, Beijing, 100069 China; 2 Department of Infectious Disease, Lanzhou Pulmonary Hospital, Lanzhou, 730046 China

**Keywords:** Adverse events, China, Antiretroviral therapy, HIV

## Abstract

**Background:**

Although the global human immunodeficiency virus (HIV) epidemic has improved significantly due to antiretroviral treatment (ART), ART-related adverse events (AEs) remain an issue. Therefore, investigating the factors associated with ART-related AEs may provide vital information for monitoring risks.

**Methods:**

A prospective cohort study was conducted among adult patients (aged 18 years or older) with HIV who received Tenofovir (TDF) + Lamivudine (3TC) + Efavirenz (EFV) as first-line ART regimens. All AEs during the first 12 months of therapy were recorded. Logistic regression analysis was used to identify variables associated with AEs.

**Results:**

Four hundred seventy-four patients receiving TDF+ 3TC+ EFV ART regimens between March 2017 and October 2017 were included in the study analysis. Among them, 472 (99.6%) experienced at least one AE, 436 (92.0%) patients experienced at least one AE within 1 month of treatment, 33 (7.0%) between one and 3 months of treatment, and three (0.6%) patients after 3 months of treatment. The most commonly reported AE was nervous system (95.6%) related, followed by dyslipidemia (79.3%), and impaired liver function (48.1%). Patients with baseline body mass index (BMI) greater than 24 kg/m^2^ (adjusted OR 1.77, 95%CI 1.03–3.02), pre-existing multiple AEs (adjusted OR 2.72, 95%CI 1.59–4.64), and pre-existing severe AEs (adjusted OR 5.58, 95%CI 2.65–11.73) were at increased odds of developing a severe AE. Patients with baseline BMI greater than 24 kg/m^2^ (adjusted OR 2.72, 95%CI 1.25–5.89) were more likely to develop multiple AEs.

**Conclusion:**

The incidence of ART-related adverse events over a 12-month period in China was high. Baseline BMI greater than 24 kg/m^2^, pre-existing multiple AEs, and pre-existing severe AEs were shown to be independent risk factors for developing a severe AE.

## Background

According to the World Health Organization (WHO), approximately 37.9 million people were living with human immunodeficiency virus (HIV) by the end of 2018, with 1.7 million people becoming newly infected in 2017, globally [1]. Among them, 23.3 million people were on antiretroviral therapy (ART) treatment by the end of 2018 [1]. With the advent of ART over the last two decades, HIV has shifted from what was once a death sentence to a treatable, chronic condition. As a result, China launched its National Free Antiretroviral Treatment Program in 2002. Since that time, more than 220,000 people living with HIV have received free ART, markedly reducing the associated morbidity and mortality of patients living with HIV and acquired immune deficiency syndrome (AIDS) [2].

In general practice, two nucleoside reverse transcriptase inhibitors (NRTIs) and an integrase strand transfer inhibitor (INSTI) are recommended as the preferred first-line ART regimens for patients living with HIV [3–5]. Non-nucleoside reverse transcriptase inhibitors (NNRTIs), such as Efavirenz (EFV), are no longer recommended as first-line therapy in developed countries due to their associated increasing levels of pre-treatment resistance and adverse events (AEs) [3–5]. However, EFV is still widely used in developing countries [5]. Under the National Free Antiretroviral Treatment Program in China, 604,160 adults were receiving ART by the end of 2017 [6], where a combination of Tenofovir (TDF), Lamivudine (3TC) and EFV are used as the first line antiviral regimen. Though ART contributes to improved survival and quality of life, adverse events associated with antiviral drugs should not be ignored [7]. AEs may lead to the discontinuation of ART, which in turn may cause therapeutic failure, treatment modifications, and consequently contribute to increased HIV virus resistance and poorer health [8]. Therefore, it is critical to find factors associated with the development of ART-associated AEs. Though several studies have assessed such risk factors in Western and African populations, little evidence exit in China. This study aimed to identify factors associated with first line ART regimen (TDF + 3TC + EFV)-related adverse events in order to better inform national treatment strategies and improve care for people living with HIV.

## Methods

### Study design and population

This was a prospective study from March 2017 to October 2017 in Beijing You’an Hospital, Capital Medical University in Beijing, China. You’an Hospital is one of the largest infectious disease hospitals in China, treating more than 9000 HIV-positive individuals. Individuals with confirmed HIV diagnosis, aged 18 years or older, whom were receiving TDF + 3TC + EFV as their first-line ART regimen were included in the study. People living with HIV who experienced severe opportunistic infections requiring hospitalization and pregnant women were excluded.

### Patient and public involvement

All participants provided written, informed consent prior to enrollment in the study authorizing the use of their data generated from the medical information system. The research question and outcome measures were explained to each participant. Patients themselves were not involved in the recruitment and conduct of the study. This study was approved by the Beijing You’an Hospital Ethics Committee (No. LL-2019-038-K).

### Follow-up

Each individual was followed at 2 weeks, 1 month, 2 months, 3 months, 6 months, 9 months, and 12 months after initiation of ART treatment. At baseline, each participant received a series of medical examinations including liver and renal function tests, routine blood work, HBV, HCV, and syphilis tests, lipid profile evaluations, HIV viral load measurements, drug resistance assessments, and CD4 cells count. Participants also completed a standard questionnaire administered by trained researchers in order to collect information on demographic and socioeconomic factors. Sleep quality statistics were measured using the Pittsburgh Sleep Quality Index (PSQI) [9]. Anxiety and depression were measured according to the Hospital Anxiety and Depression scale (HAD) [10]. Data related to liver function, renal function, routine blood work, anxiety scale, and depression scale were collected at 2 weeks, 1 month, 2 months, 3 months, 6 months, 9 months, and 12 months after initiation of treatment. Blood lipid levels were also collected at 3 months, 6 months, 9 months, and 12 months after initiation of treatment. CD4 cell count was measured at three and 12 months after initiation of treatment. Participant’s viral load was measured at six and 12 months after initiation of treatment, following national guidelines.

### Definition and categorization of adverse events

Adverse events were defined as a response to ART therapies which are noxious and unintended [11]. The diagnosis and classification of AEs were determined by combining laboratory abnormalities with symptoms reported by the participants. These were then compared using the clinical definitions outlined in the Division of Acquired Immuno Deficiency Syndrome (DAIDS) Table for Grading the Severity of Adult and Pediatric Adverse Events (DAIDS 2017) [12]. The severity of AEs was graded as follows: mild (grade 1), moderate (grade 2), severe (grade 3), life-threatening (grade 4), and death (grade 5) [12]. The cut-off values for different AE parameters are presented in Table S1 in the supplementary file.

We assessed sleep quality using the Chinese version of the Pittsburgh Sleep Quality Index (PSQI). The sleep quality scores range from 0 to 21, with a PSQI > 5 indicating some level of sleep disturbance, and a PSQI of > 10 indicating severe sleep disturbance [9, 13]. Anxiety and depression were assessed using the Hospital Anxiety and Depression scale (HADS) [14]. The associated score represents differing severity of anxiety or depression. The scale ranges from 0 to 7 (no anxiety or depression); 8–10 (mild anxiety or depression); 11–14 (moderate anxiety or depression); and 15–21 (severe anxiety or depression) [15].

A participant was classified as having multiple AEs if he or she experienced more than one of the following abnormal symptoms: liver function abnormality [Alanine aminotransferase (ALT), Aspartate aminotransferase (AST), total bilirubin (TBIL)], impaired renal function [estimated glomerular filtration rate (eGFR)], dyslipidemia [total cholesterol (TC), triglyceride (TG), low density lipoprotein cholesterol (LDL-c)], erythrism, and neuropsychiatric adverse events (NPAEs), such as headache, insomnia, anxiety, or depression). The severe AEs were defined as grades 3 and 4 of the aforementioned parameters (Table S1).

### Statistical analysis

All statistical analyses were conducted using R version 3.3.2. (R Core Team, Vienna, Austria), and two-sided *P* values < 0.05 were considered statistically significant. Chi square test (for categorical variables) and analysis of variance (ANOVA, for continuous variables) were used to compare the baseline characteristics. Univariable logistic regression model was used to identify variables associated with AEs. Variables with a *P* ≤ 0.2 in univariable analysis were further assessed with a multivariable logistic regression model.

## Results

### Incidence of drug discontinuation

In this study, 504 patients were initially included. Of them, 474 patients (94.0%) completed 12 months of follow-up and were included in the final analysis. A total of 30 (6.0%) patients discontinued treatment, 16 (3.2%) of which were due to drug-related side effects, 5 (1.0%) were due to EFV resistance, and 9 (1.8%) were due to other causes. Of the 16 patients who discontinued treatment due to any adverse event, 5 discontinued due to NPAEs (1.0%), one discontinued due to rash (0.2%), five discontinued due to abnormal liver function (1.0%), two discontinued due to abnormal renal function (0.4%), and three discontinued due to dyslipidemia (0.6%). Of those who discontinued, two discontinued between 0 to 2 weeks after ART initiation (0.5 month), 12 at one to 2 months after starting treatment, and two at three to 6 months after EFV treatment.

### Baseline characteristics

The baseline demographic characteristics of the participants are presented in Table 1. From March 2017 to October 2017, 474 patients receiving TDF + 3TC + EFV first-line ART regimens were included in the analysis. The mean age of those initiating ART was 34.0 ± 10.1 years. 193 (40.7%) participants were aged between 18 and 29 years. Four hundred sixty-three participants (97.7%) were male, 311 (65.6%) had an educational level of high school of higher.

**Table 1:** Demographic Characteristics of Participants

Variables	Mean ± SD /N(%)
**Sex**	
Female	11 (2.3%)
Male	463 (97.7%)
Age, years	34.0 ± 10.1
18–29	193 (40.7%)
30–39	168 (35.4%)
40–49	67 (14.1%)
≥ 50	46 (9.7%)
Marital status	
Married/common law marriage	107 (22.6%)
Single/divorced/widowed	367 (77.4%)
Years of Education	
≤ 9 years	88 (18.6%)
10–12 years	75 (15.8%)
≥ 13 years	311 (65.6%)
Route of HIV infection	
Homosexual transmission	427 (90.1%)
Heterosexual transmission	26 (5.5%)
Intravenous drug use	0 (0%)
Others	21 (4.4%)
BMI, kg/m^2^	22.3 ± 3.32
< 18.5	47 (9.9%)
18.5–23.9	304 (64.1%)
24–27.9	94 (19.8%)
≥ 28	29 (6.1%)
CD4+ cells/mm^3^	340.7 ± 241.2
≤ 199	119 (25.1%)
200–349	146 (30.8%)
350–499	124 (26.2%)
≥ 500	85 (17.9%)
Viral load, copies/ml	
< 100,000	382 (80.6%)
≥ 100,000	92 (19.4%)
Hepatitis B status	
Positive	31 (6.5%)
Negative	443 (93.5%)
Hepatitis C status	
Positive	5 (1.1%)
Negative	469 (98.9%)
Tuberculosis	
Yes	4 (0.8%)
No	470 (99.2%)
Syphilis	
Yes	149 (31.4%)
No	325 (68.6%)
WHO Clinical Stage	
I + II	433 (91.4%)
III + IV	41 (8.6%)

### Incidence of adverse events

The frequency and type of adverse events are shown in Table 2. Of the 474 patients, 472 (99.6%) of them experienced at least one AE, of which 387 (82.0%) were classified as mild or moderate, while 85 (18.0%) were classified as severe or life-threatening. Four hundred sixty-three patients (97.7%) experienced at least one AE within 3 months of initiating treatment, while 342 patients (72.2%) experienced at least one AE 3 months after starting treatment. The most commonly reported AEs were NPAEs (95.6%), followed by dyslipidemia (79.3%), liver function abnormalities (48.1%), skin lesions (11.6%), and renal problems (6.5%). Among severe AEs, the most prominent were increased dyslipidemia (10.1%), NPAEs (4.6%), impaired liver function (4.2%), renal problems (0.4%), and skin lesions (0.2%).

**Table 2:** Frequency of the most common adverse events

	Baseline			After treatment		
Adverse Event	Grade 1–2	Grade 3–4	Total	Grade 1–2	Grade 3–4	Total
Liver function	97(20.5%)	2(0.4%)	99(20.9%)	208 (43.9%)	20 (4.2%)	228 (48.1%)
ALT	31(6.5%)	1(0.2%)	32(6.8%)	181 (38.2%)	14 (3.0%)	195 (41.1%)
AST	63(13.3%)	0(0%)	63(13.3%)	144 (30.4%)	13 (2.7%)	157 (33.1%)
TBIL	33(7%)	1(0.2%)	34(7.2%)	17 (3.6%)	3 (0.6%)	20 (4.2%)
Impaired renal function						
eGFR	6(1.3%)	1(0.2%)	7(1.5%)	29 (6.1%)	2 (0.4%)	31 (6.5%)
Dyslipidemia	116(24.4%)	7(1.5%)	123(25.9%)	328 (69.2%)	48 (10.1%)	376 (79.3%)
TC	31(6.5%)	0(0%)	31(6.5%)	253 (53.4%)	8 (1.7%)	261 (55.1%)
TG	92(19.4%)	3(0.6%)	95(20%)	265 (55.9%)	38 (8.0%)	303 (63.9%)
LDL-c	24(5.1%)	4(0.8%)	28(5.9%)	112 (23.6%)	13 (2.7%)	125 (26.4%)
Skin lesions						
Erythra	34(7.2%)	1(0.2%)	35(7.4%)	54 (11.4%)	1 (0.2%)	55 (11.6%)
Nervous system	328(69.2%)	25(5.3%)	353(74.5%)	431 (90.9%)	22 (4.6%)	453 (95.6%)
Headache	44(9.3%)	0(0%)	44(9.3%)	51 (10.8%)	0 (0%)	51 (10.8%)
Insomnia	321(67.7%)	0(0%)	321(67.7%)	446 (94.1%)	0 (0%)	446 (94.1%)
Anxiety	115(24.3%)	19(4%)	134(28.3%)	192 (40.5%)	14 (3.0%)	206 (43.5%)
Depression	92(19.4%)	12(2.5%)	104(21.9%)	176 (37.1%)	14 (3.0%)	190 (40.1%)
Total	367(77.4%)	36(7.6%)	403(85.0%)	387 (81.7%)	85 (17.9%)	472 (99.6%)

### Predictors of adverse events

Patients with baseline BMI greater than 24 kg/m^2^ (adjusted OR 1.77, 95%CI 1.03–3.02), pre-existing multiple AEs (adjusted OR 2.72, 95%CI 1.59–4.64), and pre-existing severe AE (adjusted OR 5.58, 95%CI 2.65–11.73) were at increased odds of developing a severe AE (Table 3). Age, marital status, education, CD4 count, viral load, syphilis status, hepatitis status, WHO clinical stage at baseline, and pre-existing severe AEs were not significantly associated with incident severe AE. Patients with baseline BMI greater than 24 kg/m^2^ (adjusted OR 2.72, 95%CI 1.25–5.89) were at increased odds of developing multiple AE (Table 4). Age, marital status, education, CD4 count, viral load, syphilis status, hepatitis status, and WHO clinical stage, and pre-existing severe AEs were not significantly associated with developing multiple AEs.

**Table 3:** Socio-demographic and clinical factors associated with severe adverse events

Characteristic at baseline	Case/n	Crude model		Adjusted model	
		OR(95%CI)	*P*	OR(95%CI)	*P*
Age, years					
< 50	77/428	1			
≥ 50	8/46	0.96(0.43–2.14)	0.920		
Marital status					
Single/divorced/widowed	63/367	0.80(0.47–1.38)	0.421		
Married/common law marriage	22/107	1			
Years of Education					
≤ 9	18/88	1.22(0.69–2.19)	0.495		
> 9	67/386	1			
BMI, kg/m^2^					
< 24	54/351	1		1	
≥ 24	31/123	1.85(1.12–3.06)	0.016	1.77(1.03–3.02)	0.038
CD4+, cells/mm^3^					
≤ 199	21/120	1			
200–349	22/145	0.84(0.44–1.62)	0.843		
350–499	24/124	1.13(0.59–2.16)	0.656		
≥ 500	18/85	1.27(0.63–2.56)	0.509		
Viral load, cells/ml					
< 100,000	64/382	1		1	
≥ 100,000	21/92	1.47(0.84–2.56)	0.175	1.55(0.86–2.81)	0.148
Syphilis Status					
Yes	24/149	0.83(0.50–1.40)	0.483		
No	61/325	1			
Hepatitis Status					
Positive	7/31	1.37(0.57–3.28)	0.487		
Negative	78/443	1			
WHO Clinical Stage					
I + II	75/433	1			
III + IV	10/41	1.54(0.72–3.28)	0.262		
Pre-existing AE					
Yes	78/403	2.19(0.97–4.97)	0.060	0.96(0.39–2.34)	0.924
No	7/71	1		1	
Pre-existing multiple AE					
Yes	49/167	3.13(1.93–5.06)	< 0.001	2.72(1.59–4.64)	< 0.001
No	36/307	1		1	
Pre-existing severe AE					
Yes	18/36	5.54(2.74–11.19)	< 0.001	5.58(2.65–11.73)	< 0.001
No	67/438	1		1

**Table 4:** Socio-demographic and clinical factors associated with multiple adverse events

Characteristic at baseline	Case/n	Crude model		Adjusted model	
		OR(95%CI)	*P*	OR(95%CI)	*P*
Age, years					
< 50	364/428	1		2.54(0.76–8.51)	0.130
≥ 50	43/46	2.52(0.76–8.37)	0.131	1	
Marital status					
Single/divorced/widowed	314/367	0.89(0.47–1.68)	0.723		
Married/common law marriage	93/107	1			
Years of Education					
≤ 9	75/88	0.94(0.49–1.81)	0.849		
> 9	332/386	1			
BMI, kg/m^2^					
< 24	292/351	1		1	
≥ 24	115/123	2.91(1.35–6.27)	0.007	2.72(1.25–5.89)	0.012
CD4+, cells/mm^3^					
≤ 199	102/120	1			
200–349	119/145	0.81(0.421.56)	0.524		
350–499	110/124	1.39(0.66–2.93)	0.392		
≥ 500	76/85	1.49(0.64–3.50)	0.360		
Viral load, cells/ml					
< 100,000	327/382	1			
≥ 100,000	80/92	1.12(0.57–2.19)	0.738		
Syphilis Status					
Yes	129/149	1.09(0.62–1.92)	0.763		
No	278/325	1			
Hepatitis Status					
Positive	25/31	0.67(0.26–1.69)	0.391		
Negative	382/443	1			
WHO Clinical Stage					
I + II	374/433	1			
III + IV	33/41	0.65(0.29–1.48)	0.651		
Pre-existing AE					
Yes	346/403	1.00(0.48–2.06)	0.989		
No	61/71	1			
Pre-existing multiple AE					
Yes	150/167	1.72(0.96–3.08)	0.071	1.57(0.86–2.84)	0.139
No	257/307	1		1	
Pre-existing severe AE					
Yes	33/36	1.88(0.56–6.32)	0.306		
No	374/438	1			

## Discussion

This is the first prospective cohort study to assess the incidence, type, severity, and predictors of adverse events in patients who were initiated on TDF + 3TC + EFV ART in China. We found that almost 100% of patients experienced at least one adverse event, while approximately 20% experienced at least one severe AE. The most common severe adverse events in this study were NPAEs, dyslipidemia, and liver function abnormalities. Participants whose baseline BMI was greater than 24 kg/m^2^, had pre-existing multiple AEs, and pre-existing severe AEs, were shown to be at increased odds for developing a severe AE. Our findings regarding the risk factors for AEs (i.e. BMI greater than 24 kg/m^2^, pre-existing multiple AEs, and pre-existing severe AEs) are important, as they suggest that practical interventions to correct these factors could be implemented in clinical practice in order to reduce the risk of an incident AE.

In this study, 99.6% of patients experienced at least one AE, which is higher than what has been reported in Brazil (33.6%) [16], Aferica (40.3/1000 person-years) [17], and India (32.45%) [18]. The high incidence of AE in this study might be due to the prospective nature of the study, which could contribute to the identification of mild AEs that might be unfounded in other analyses. What’s more, HIV infection is regarded as a traumatic and stressful experience; patients with HIV infection are more likely to exhibit mental health problems than the general population, especially soon after diagnosis [19–21]. Compared with other countries or regions, a systematic review in China revealed a higher prevalence of depression (greater than 60%) and anxiety (greater than 40%) in patients with HIV infection [22], which may have led to an increase NPAEs. In addition, most patients in the current study experienced at least one pre-existing severe AE, which might increase the risk of incident AEs. In addition, previous studies suggested that CYP2B6 516 polymorphisms is a major enzyme in the EFV metabolic pathway. However, Chen et.al reported that the allelic frequency of CYP2B6 polymorphisms in Chinese population is 0.16 [23], which is different from other populations [24].

Previous studies have demonstrated that Efavirenz-based regimens can lead to adverse effects impacting the central nervous system [25]. Consistently, nervous system AEs were the most commonly reported AE in our study. Studies evaluating the safety of EFV/TDF/3TC single-tablet regimens have also reported a neuropsychiatric adverse event profile similar to that found in this study [26, 27]. Other research has found that patients using EFV combined with TDF and Emtricitabine (FTC) had higher occurrences of neuropsychiatric events than those using Dolutegravir (DTG) in combination with Abacavir (ABC) and 3TC [28]. We also reported a much higher frequency of NPAEs (95.5% of patients) than a previous review [29]. With the exception of dizziness, fewer than 10% of patients exposed to EFV experienced any other specific type of neuropsychiatric event. This may be because the patients in our study were enrolled soon after their HIV diagnosis, with 70.0% of the patients initiating ART less than 1 month after diagnosis. This may have resulted in the higher baseline abnormal nervous system rate. However, there were no other increases in grade 3–4 NPAEs after 1 year of treatment.

Previous studies reported that patients experiencing severe AEs were more likely to have a lower BMI [8, 30]. One such study in Ghana found that patients with BMI < 16 kg/m^2^ had increased risk of neuropsychiatric toxicity (aHR: 1.44; 95% CI: 1.02–2.03) [31], perhaps due to higher blood concentration of EFV, which is expected in underweight people [32]. However, we found that the risk of AE may increase with increased BMI. This discrepancy might be because the Ghana study focused only on EFV neuropsychiatric toxicity, including insomnia (50%), headaches (8%), dizziness (7%), and abnormal dreams (6%). In our study, we described all major AEs including liver function abnormalities, impaired renal function, dyslipidemia, erythrism, and neuropsychiatric adverse events. Of these, dyslipidemia and liver function abnormalities were believed to be related with higher BMI. When only considering neuropsychiatric adverse events, there was no relation with BMI (data not shown). Additionally, participants in the Ghana study had much lower BMI than those in our study [median (IQR): 22.0 (20.1–24.1) kg/m^2^ for current study vs. 20.0 (18.0–22.0) kg/m^2^ for the Ghana study]. Lastly, we found that patients who had at least one pre-existing severe AE were at increased odds of experiencing an adverse event, signifying that patients with pre-existing AEs will need tailored care in order to prevent the occurrence of AEs.

The relationship between CD4+ count and AEs is inconsistent. Khalili et al., previously reported that there was no significant association between CD4+ cell count and developing an adverse event [33], which is consistent with findings from our study. Lartey et al., however, reported that patients with CD4+ cell counts higher than 250 cells/mm3 had a higher chance of developing an AE [34]. Mendes et al., also reported that higher CD4+ cell counts were associated with lower risk of incident AE [35]. This difference might be due to the different types of AEs reported and different antiretroviral regimens used between studies.

This study has several important strengths. First, the occurrence of AE was defined according to active surveillance of laboratory and clinical parameters. Second, the prospective design of the study makes it possible to detect the temporal relationship between risk factors and development of an AE. Lastly, we investigated the predictors for various AEs. Despite these strengths, we should also acknowledge several limitations. First, it is possible that there were unmeasured confounders that could affect these associations. Second, participants in our study were from a single center (the Beijing You’an Hospital), thus our results should be generalized with caution.

## Conclusions

Almost 100% of patients who were initiated on TDF + 3TC + EFV first-line ART in China experienced adverse events. Baseline BMI greater than 24 kg/m^2^ and pre-existing severe AEs were independent risk factors for experiencing a severe adverse event. Further studies with larger sample sizes are needed to increase awareness of the frequency and types of AE associated with the use of antiretrovirals in China.

## Supplementary information

**Additional file 1: Table S1.** Definition of adverse events

## Data Availability

The datasets used and/or analyzed during the current study are available from the corresponding author upon reasonable request.

## Abbreviations

3TC: Lamivudine
ABC: Abacavir
AEs: Adverse events
AIDS: Acquired immune deficiency syndrome
ART: Antiretroviral treatment
BMI: Body mass index
DAIDS: Division of Acquired Immunodeficiency Deficiency Syndrome
DTG: Dolutegravir
EFV: Efavirenz
HAD: Hospital anxiety and depression scale
HIV: Human immunodeficiency virus
INSTI: Integrase strand transfer inhibitor
NNRTIs: Non-nucleoside reverse transcriptase inhibitors
NRTIs: Nucleoside reverse transcriptase inhibitors
PSQI: Pittsburgh sleep quality index
TDF: Tenofovir
