# Supplementary material for: Adverse events in Chinese human immunodeficiency virus (HIV) patients receiving first line antiretroviral therapy
Source: BMC Infect Dis. 2020 Feb 19;20:158. doi: 10.1186/s12879-020-4878-2 (PMC7031902; doi:10.1186/s12879-020-4878-2)
Supplement: Supplementary file 1 — Additional file 1: Table S1. Definition of adverse events [file 12879_2020_4878_MOESM1_ESM.docx]

**Table S1.** Definition of adverse events

| Parameter | Mild  (grade 1) | Moderate  (grade 2) | Severe  (grade 3) | Life-threatening  (grade 4) |
| --- | --- | --- | --- | --- |
| ALT (ULN) | 1.26-2.50 | 2.6-5.0 | 5.1-10.0 | ＞10.0 |
| AST | 1.26-2.50 | 2.6-5.0 | 5.1-10.0 | ＞10.0 |
| TBIL | 1.26-2.50 | 2.6-5.0 | 5.1-10.0 | ＞10.0 |
| eGFR(mL/min/1) | 60-90 | 30-60 | 15-30 | <15 |
| TC (mmol/L) | 5.18-6.19 | 6.19-7.77 | ≥7.77 | N/A |
| TG (mmol/L) | 1.71-3.42 | 3.42-5.7 | 5.7-11.4 | >11.4 |
| LDL (mmol/L) | 3.17-4.12 | 4.12-4.90 | ≥4.90 | N/A |
| Erythrism | Pruritus erythema | Diffuse maculopapulation or desquamation | Blister or wet desquamation ulcer | Suspected Stevens Johnson syndrome, necrotizing exfoliative dermatitis |
| Headache | Symptoms causing no or minimal interference with usual social & functional activities | Symptoms causing greater than minimal interference with usual social & functional activities | Symptoms causing inability to perform usual social & functional activities | Symptoms causing inability to perform  basic self-care functions OR Hospitalization indicated OR Headache with significant impairment of alertness or other neurologic function |
| Insomnia (PSQI score) | 6-11 | 11-21 | N/A | N/A |
| Anxiety (HAD score for anxiety) | 8-10 | 11-14 | 15-21 | N/A |
| Depression (HAD score for depression) | 8-10 | 11-14 | 15-21 | N/A |
